# A New Perspective on Delusional States – Evidence for Claustrum Involvement

**DOI:** 10.3389/fpsyt.2015.00158

**Published:** 2015-11-09

**Authors:** Maria Cristina Patru, David H. Reser

**Affiliations:** ^1^Department of Psychiatry, Hôpitaux Universitaires de Genève, Geneve, Switzerland; ^2^Department of Physiology, Monash University, Melbourne, Australia

**Keywords:** claustrum, delusions, positive symptoms, psychosis, mesolimbic dopamine system

## Abstract

Delusions are a hallmark positive symptom of schizophrenia, although they are also associated with a wide variety of other psychiatric and neurological disorders. The heterogeneity of clinical presentation and underlying disease, along with a lack of experimental animal models, make delusions exceptionally difficult to study in isolation, either in schizophrenia or other diseases. To date, no detailed studies have focused specifically on the neural mechanisms of delusion, although some studies have reported characteristic activation of specific brain areas or networks associated with them. Here, we present a novel hypothesis and extant supporting evidence implicating the claustrum, a relatively poorly understood forebrain nucleus, as a potential common center for delusional states.

## Introduction

Delusions are broadly categorized as a misapprehension of reality, which has a realistic character for the patient, and to which he or she adheres with conviction despite contradiction by reality, experience, or collective beliefs. Delusions are of particular diagnostic importance in schizophrenia, and current treatments, including antipsychotics, exhibit variable effectiveness against them (1, 2). Therapies for delusions that occur in non-schizophrenic psychiatric (e.g., bipolar disorder, depression, dementia, substance use) and neurological disorders (e.g., tumors, infectious, metabolic, traumatic, vascular, autoimmune disorders) are similar to those used to treat schizophrenia (e.g., antipsychotics), and exhibit similar levels of efficacy in all these disorders.

Delusions are a heterogeneous concept, with several semiotic entities (delusional feelings or mood, delusional perceptions, and delusional thoughts), systematization degrees (organization in a logical manner, with causal links, or not), modalities of elaboration (interpretative, imaginative, intuitive, or hallucinatory) and themes (delusions of reference, persecution, jealousy, guilt, hypochondriacal, grandeur, and so on). Varying degrees of delusional ideation are also found in the healthy population (3–6).

Although some experimental animal models of psychotic behavior are available [e.g., see Ref. (7)], it is nearly impossible to establish a concrete link between measures of overt responses in animals, such as paired pulse inhibition, conditioned avoidance, or latent inhibition, and changes in abstract representation of the external world by an individual (8). These limitations have led to slow progress in research surrounding delusions.

Despite numerous studies of patients with psychotic symptoms describing characteristic activation of specific brain areas or networks (9–17), there have been no detailed studies to date focusing specifically on delusions and their neural mechanisms. Here, we present a review of evidence obtained from a wide range of studies, employing a diverse array of analytical approaches, which has led to speculate that *the claustrum, a relatively poorly understood forebrain nucleus, may be a critical link in the chain of causality for delusional states*. Suggestive evidence of structural and/or functional changes in the claustrum as a component of delusions, or psychotic symptoms more generally, has been widely reported. However, due to sparse information about the functional organization of the claustrum, and the difficulty of studying this nucleus in isolation, causality cannot be inferred from the existing literature. Thus, the proposed hypothesis necessarily rests upon indirect, and in some cases, non-specific evidence. Our purpose in this review is to familiarize clinicians and researchers with the current (relatively poor) state of understanding of claustrum structure and function, and to introduce testable and accessible research questions that could be employed to evaluate this hypothesis.

**Figure 1:**
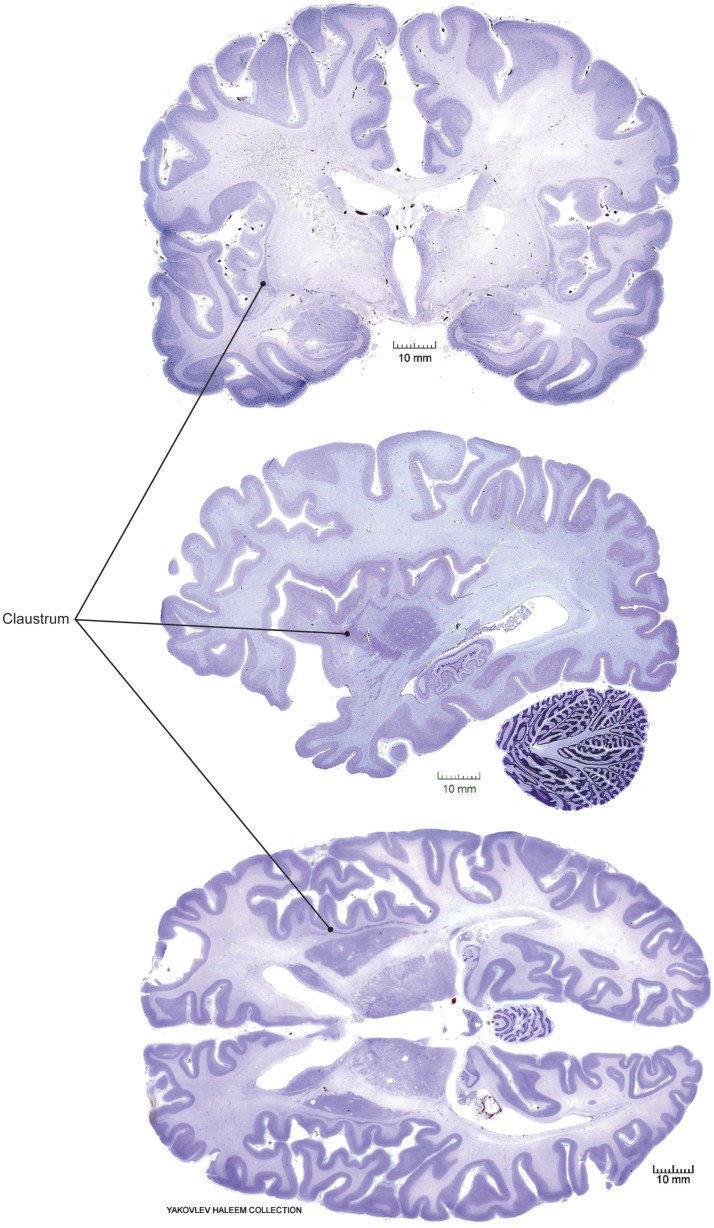
**Claustrum location in human brain**. Coronal (top), sagittal (middle), and horizontal (bottom) views from Nissl-stained postmortem sections obtained from a public repository. Solid circles indicate the position of the claustrum in each view. Note, sections are not taken from the same three-dimensional position, i.e., sections are not registered with respect to the whole brain structure. Images adapted with permission from http://www.brains.rad.msu.edu, and http://brainmuseum.org, supported by the US National Science Foundation.

The claustrum is a deep gray matter brain structure, first identified in the eighteenth century (18). Many researchers have since commented on its unusual anatomy, ontogeny, histology, vasculature, and cytochemistry. However, the function or functions of the claustrum remain uncertain (19). The convoluted shape and relatively inaccessible location of the claustrum render it difficult to study using conventional methods, though recent advances in structural and functional imaging have identified claustrum dysfunction in a number of psychiatric symptoms and diseases. In primates, the claustrum is thin in cross section, but broad and elongated along the rostral–caudal and dorsal–ventral axes (Figure 1). Targeted lesions, microinjections of tracer or drugs, and placement of recording electrodes for electrophysiology are complicated by the virtual certainty of injury to adjacent structures, including the insular cortex, external and extreme capsules, and striatum.

Synthesis of knowledge about possible relationships between the claustrum and delusions is complicated by several factors, including (1) the relative paucity of studies that focus on the claustrum, as opposed to its inclusion in a long list of “accessory structures,” which exhibit changes in a given condition; (2) the even larger number of functional studies in which the claustrum is subsumed in activity assigned to the anterior insula and/or striatum; and (3) the lack of a clear framework for claustrum function in normal behavior [reviewed in Smythies et al. (20)]. All of the major current hypotheses regarding claustrum function, described in detail in Box 1, involve mental processes that are essential for accurate interpretation of sensory information in the context of past experiences. This “reality testing” function is compromised across delusions, regardless of the modality or specific features of the delusion.

Box 1 **Recent hypotheses surrounding claustrum function(s).**

Here, we describe the major working hypotheses regarding claustrum function that have been published in the past decade. We recognize (1) that there is considerable overlap in the roles proposed here, (2) that these hypotheses are not mutually exclusive, and (3) that all of these proposals may well be incomplete, or flat wrong. However, these models underpin much of the current experimental work surrounding the neuroanatomical, neurophysiological, and neurochemical properties of the claustrum, and delusional symptoms could arise from dysfunction of any of these processes.

1. The “orchestra conductor” – first proposed by Crick and Koch (21), this influential hypothesis suggests that the claustrum acts to synchronize activity across the various timescales of sensory modalities, allowing for generation of a unified percept of the external world (sensory binding). The model rests on the central location of the claustrum in each cerebral hemisphere, as well as its high intrinsic connectivity and fairly uniform cytoarchitecture, all of which could act to smooth out disparities in temporal activity occurring within each modality that result from the same environmental source, such as the size and direction of motion (visual), hoofbeats (auditory), and vibration (somatosensory) of a charging bull, allowing it to be perceived as a single object.
2. Sensory integration/coincidence detection – proposed by Smythies et al. (22), this hypothesis has undergone several refinements. In its most recent iteration, the claustrum functions as a coincidence or mismatch detector, by means of amplification of oscillatory activity in reverberating claustro-cortical circuits. Oscillatory activity generated in disparate cortical or subcortical regions could presumably be reinforced, either enhancing the perceptual salience of a target, or as a function of target salience (causality is not yet clear in this case).
3. Modulation/switching of cortical functional networks – First outlined by Reser et al. (23), this hypothesis is based on the connectional anatomy of claustro-cortical circuits in the prefrontal cortex (PFC) of the monkey. Recent work (24), has shown that the transition between increased synchrony of the (task negative) default mode, and (task positive) central executive network is likely mediated by activation of a third network, the salience network described by Seeley et al. (25). This switching role for the salience network has recently been confirmed by Goulden et al. (26), and the area templates used for both the salience and central executive networks in that study clearly overlap the claustrum, especially in the left hemisphere [(26), their Figure 1]. Prefrontal areas involved in both the default mode and central executive networks exhibit overlapping claustrum representations, and it is interesting to note that stimulus salience also figures prominently in hypotheses 2 (above), and 4 (below). Note that imaging studies published to date have ascribed activity in the salience network to the insular cortex (immediately adjacent to the claustrum), rather than the claustrum. Full explication of this disparity is outside the scope of this work, but the connectional anatomy of New World monkey species suggest that the claustrum is a plausible hub for this network.
4. Modulation of selective attention – Recent theoretical work and review of experimental data led Goll et al. (27) to propose that the claustrum is a key locus for modulation of selective attention. Crucially, their model incorporates both the laminar circuitry of the cerebral cortex and the modulation of claustro-cortical circuits via clearly described subcortical pathways, which facilitates experimental testing of the model. In particular, their description of the putative circuitry in the rodent brain will allow for modern molecular and genetic tools to be applied to the claustrum in ways not previously feasible.

Here, we hypothesize a role for claustrum dysfunction in the pathophysiology of delusions across several psychiatric disorders. This hypothesis is based on the widespread cortical connectivity of the claustrum and on the common feature of delusional symptoms, which is impairment of the ability to accurately assess the “validity” of perceptual judgments regarding mental representations of the external world. Breakdown of this process permits patients to accept, even embrace, constructs of reality that contain implausible, or even bizarre characterizations.

## Structural Connectivity, Electrophysiological, and Functional Studies of the Claustrum

A prominent feature of the claustrum in all species studied to date is its pervasive and reciprocal connectivity with most areas of the cerebral cortex. In both rodents and humans, the claustrum is among the most connected forebrain structures in the brain (28), with extensive cortical and subcortical projections. The organization of structural connections as defined by tracer and/or electrophysiological studies in cats and monkeys shows both rostro-caudal and medio-dorsal topography of connections with sensory cortex. In the visual domain, retinotopic connections with striate cortex are observed in dorsal–caudal claustrum (29, 30), while auditory representation [mid and caudal-ventral, (31)] and somatomotor representations (32) have also been described. Extensive projections to the PFC have also been reported, predominantly localized in the mid-ventral and rostral claustrum [(23); reviewed in Druga (33)].

In a detailed microdissection and tractography study of postmortem human brains, Fernandez-Miranda et al. (34) identified two prominent projection pathways emanating from the claustrum: a dorsal pathway containing the bulk of the fibers connected with the sensory and motor areas of cortex, and a ventral pathway containing uncinate fasciculus fibers, which transit and merge with the ventral claustrum gray matter. Interestingly, that study also reported that cortico-claustral fibers account for the majority of axons in the external capsule, with a much smaller group connecting with the putamen. These claustro-cortical fibers also exhibited spatial topography, with the caudal portions of cortex connected with the more caudal section of the dorsal claustrum, and the rostral cortical connections comprising the anterior external capsule fibers and anterior dorsal claustrum tracts. Tractography studies in humans largely support the patterns of connectivity described above, with some refinements. Milardi et al. (35) identified four principal white matter tracts connecting the claustrum with other structures, suggesting some of these may have been unavoidably ablated by the microdissections of Fernandez-Miranda et al. The additional projection pathways identified by Milardi et al. include a medial claustrum-basal ganglia pathway and interhemispheric projections terminating in the contralateral claustrum and cortex.

Topography of claustrum-cortex projections was also reported by Morys et al. (36), based on a detailed postmortem study of nine adult brains with ulegyria (malformation characterized by localized gyral hypotrophy following ischemic injury in the neonatal period). They noted a correlation between pathological reduction in the size of the frontal lobes and the anterior claustrum, and between the reduced size of the parieto-occipital lobes and the central and posterior claustrum.

Electrophysiological studies of claustrum function are rare in the literature, which is perhaps not surprising given the difficult surgical approach and unusual geometry of this structure. Morys and colleagues (37, 38) have studied sensory evoked potentials (SEPs) in the ipsilateral cortex, both in patients with unilateral lesions exclusively of claustrum or other parts of the brain (simultaneous lesions were not observed in the studied population) and in healthy subjects. In subjects with unilateral isolated claustrum injuries, after stimulation of the median nerve, SEPs were absent from the ipsilateral cortex when controlateral claustrum was injured. This observation was not present in patients with unilateral somatosensory-parietal, somatosensory-thalamic relay nuclei, or internal capsule lesions. The authors concluded that the claustrum exerts influence upon the contralateral somatosensory cortex. We are not aware of equivalent testing performed in any other modality, so this remains an isolated finding, but recently claustro-cortical fibers crossing the midline were demonstrated in humans (35). Our observations indicate, however, that the majority of claustrum-PFC connections are ipsilateral in the primate brain (23).

In a recent and tantalizing case report, Koubeissi and colleagues described electrical stimulation mapping of the claustrum with depth electrodes in a 54-year-old female patient with intractable epilepsy [seizure origin in left amygdala; (39)]. They reported that a reversible stimulation of the left claustrum elicited interruption of awareness for the duration of the stimulation, followed by a return to normal function, comparable to an absence-type seizure. No similar effect was observed when electrodes in the adjacent insula or putamen were activated. Although restricted to a single case, this report has elicited new interest in the claustrum as a potential causal or permissive structure in the origin of conscious awareness.

## Claustrum Morphology – Considerations for Imaging Studies

A claustrum or claustrum-like structure has been described in all placental mammals studied to date [reviewed in Smythies et al. (20)]. A full comparison of the known claustrum anatomy across species is beyond the scope of this work, but we will briefly describe the prominent features of this structure, especially those that will aid in the design or interpretation of future research studies. Readers seeking more detailed exploration of the comparative aspects of claustrum functional organization are referred to the recent Frontiers in Systems Neuroscience Special Topics collection edited by Deutch and Mathur (40) http://journal.frontiersin.org/researchtopic/1745/the-claustrum-charting-a-way-forward-for-the-brains-most-mysterious-nucleus.

The claustrum sits in a notoriously inaccessible location, extending vertically like a corrugated sheet between the external and extreme capsules, in the anterior fronto-temporal region. Morphologically, the claustrum follows the concavity of the insular cortex, while its internal curvature follows the convexity of the putamen. Although the gray matter volume of this structure has been examined using imaging methods, the precise anatomical boundaries remain in dispute in both human and animal subjects (23, 41, 42). One consequence of this morphological indeterminacy is the difficulty of ascribing activity detected using functional imaging to claustrum-containing voxels, especially at the magnetic field strengths typical of current clinical imaging systems (43).

In a postmortem imaging study based on data from the Visible Human Project, the volume of the claustrum was asymmetrical, with ~15–20% greater volume in the right hemisphere (44), corresponding to an average volume of 829 mm^3^ on the right side and 706 mm^3^ on the left. This in turn corresponded to ~10% of the volume of the overlying insular cortex. Recently, Torgerson et al. (45) reported a similar degree of asymmetry (right hemisphere is ~13% less than left hemisphere volume) in an fMRI/DTI study of 100 neurologically normal subjects, though the absolute volumes reported in that study were somewhat smaller than the claustrum volume reported by Kapakin (44). Milardi and colleagues (35) reported consistently less hemispheric asymmetry in their diffusion imaging study of 10 volunteers, with the claustrum volume in male subjects closely matching the volumes reported by Kapakin (44), and the volumes of the female subjects more closely aligned with the reported volumes of Torgerson et al. (45). While these differences are most likely methodological, or due to restricted sample sizes, the variation across studies should temper interpretation of volumetric findings in pathological conditions until a better estimate of the normal claustrum volume is obtained (preferably at several different life stages).

## Functional Connectivity of the Claustrum

It is increasingly apparent that the claustrum is a hub region of one or more functional networks in the brain, and understanding its function will require emplacement in a networked context. These networks have been identified largely from synchronous oscillations of blood flow or field potential activation during awake “resting states” in which subjects are not engaged in other tasks [reviewed in Keilholz (46)]. The best characterized of these networks include the default mode network [(47); reviewed in Raichle (48)], the salience network (25, 49), and the central executive network (25, 50). The hub areas of each of these networks have dense claustrum connectivity, and importantly, homologs of the major networks have been identified in non-human primates (51, 52) and in rodents (53–55), allowing for controlled experimental investigation of network properties.

Recently, Satterthwaite and Baker (56) reviewed a growing body of evidence that is converging on abnormalities in development of these networks as a risk factor for psychosis. Specifically, they outline how failure of these networks to segregate from a highly overlapping pattern in childhood into a heterogeneous group of networks with high internal connectivity represents a characteristic pattern in adults with psychotic symptoms. Analysis of the structural connectivity of the claustrum has shown that it occupies a position within several key functional networks (45, 57), which is consistent with both the observed functional connectivity of the networks and with our speculation that damage to the claustrum could precipitate or permit acceptance of delusional thoughts (see hypotheses 3 and 4 in Box 1). In proposing this hypothesis, we acknowledge up front that disruption of these networks has been implicated in numerous other disorders, many of which do not include delusions among their symptoms, so there are obviously factors could impact the function of these networks apart from any claustrum involvement.

Using structural and diffusion tensor neuroimaging in a large sample (*N* = 100) of healthy subjects, Torgerson and colleagues found that the claustrum had the highest connectivity density among all examined brain regions (45). Graph theoretical analyses revealed that the claustrum is a primary contributor to global brain network architecture, and that significant connectivity dependencies exist between the claustrum, frontal lobe, and cingulate regions.

Recently, an analysis of over 16,000 structural connections between brain areas in rats, as identified by neuroanatomical tracer injections, included the claustrum among the three most densely connected structures in the forebrain [along with the medial and lateral subdivisions of the entorhinal cortex; (28)]. No similar analysis of tracer studies conducted in the primate claustrum is available, but it is likely that the claustrum is among the most highly connected primate brain regions as well. This rich connectivity has been the foundation of most hypotheses regarding claustrum function (20, 21, 23).

## Evidence for Claustrum Involvement in Delusional Processes

Only a few clinical studies have described the neurological lesions or conditions where the claustrum was the sole region of involvement (58, 59). This may support the idea that dysfunction in the claustrum results primarily in disruption of higher behavioral and cognitive functions associated with the functional networks described in the previous section, rather than a specific, unitary deficit (42). Consistent with this pattern, Jacobson-McEwen et al. (60) found decreased intrinsic functional connectivity between the claustrum and dorsal anterior cingulate cortex (hubs of the default mode and salience networks, respectively) in adolescents exhibiting psychotic symptoms (delusions and hallucinations). Alternatively, it is possible that isolated spontaneous lesions of the claustrum are exceedingly rare, with lesions in this area likely also to include damage to the surrounding white matter, insular cortex, and/or putamen.

Currently, only indirect evidence exists for claustrum dysfunction in delusions, due in part to the paucity of studies in human subjects that feature targeted investigation of the claustrum, and in part to lack of an explicit animal model of delusions. Volumetric studies (see Table 1) have been especially suggestive. In schizophrenia, the intensity of delusions is inversely related to the volume of the left claustrum and right insula (61). Reduced claustrum volume on the left side was also positively correlated with the presence of delusions in patients suffering from Alzheimer’s disease (62). In that study, reduced gray matter volume in the left insula was correlated with agitation, and reduced putamen volume was associated with apathy, but neither area exhibited a significant correlation with the presence of delusions. Gray matter volume reduction was observed in the insular region of patients with delusions associated with bipolar disorder (14). Our interpretation of the Radaelli et al. study suggests that there may also have been claustrum involvement in the affected region (see, e.g., their Figure 1), though the authors did not comment on this. It is of course possible that the observed gray matter reductions could be associated with other aspects of the disorder, such as mania or depression, but this is unlikely, as the retrospective study design specifically compared patients with a history of delusions to a matched clinical sample with no past delusions, rather than to a healthy control population. The authors extensively discussed the importance of dysregulation of salience processing for delusions, and importantly, the population they studied specifically excluded subjects with hallucinations. Wolf et al. (63) also reported decreased gray matter volume in the insular region, accompanied by increased white matter volume in the external and extreme capsule region, in 16 non-demented, non-schizophrenic patients exhibiting delusional infestation. Close examination of the data presented in that study suggests involvement of the claustrum as well, which would also be consistent with increased white matter volume (i.e., possibly due to loss of the intervening claustrum gray matter – see their Figure 1B, middle), though the authors again did not specifically call out the claustrum as an affected structure.

**Table 1:** **Clinical/imaging studies reporting delusions associated with volumetric or morphological changes in the claustrum**.

Reference	Study	Within study prevalence of delusions	Results
Bruen et al. (62)	*In vivo*, MRI: 31 patients/Alzheimer’s disease	16% Of patients	High delusion scores correlated significantly with low grey mater density values in: right inferior frontal gyrus; right inferior parietal lobule, left inferior and medial frontal gyri, left claustrum
Cascella et al. (61)	*In vivo*, MRI: 43 patients/schizophrenia	70% Of patients	Significant inverse correlations between ratings of the severity of delusions and volumes of: the *left claustrum and* the right insula
			No significant correlation between cerebral gray mater volume and ratings of hallucinations
Bernstein et al. (64)	Postmortem: 14 patients/schizophrenia: 15 normal control subjects	57% Of patients (8/14 patients) exhibit positive symptoms (no specific data for delusions and hallucinations frequency)	Estimated claustrum volume reductions were between 25 and 30% of controls. Moreover, when dividing the schizophrenia group (14 patients) into “paranoid schizophrenia” (8 patients) and “residual schizophrenia” (6 patients) subgroups, the significant bilateral volume reductions in schizophrenia subjects were found to be (mainly) caused by the paranoid schizophrenia group

More recently, in a postmortem study, Bernstein et al. (64) found bilaterally reduced claustral volumes associated with both schizophrenia and major depressive disorder. Although the overall number of brains examined was relatively small, especially for major depressive disorder, several interesting patterns were evident in this study. In particular, the severity of positive symptoms (delusions and hallucinations) was correlated with the degree of volume loss in the post mortem claustrum, showing that significantly smaller volumes are present in subjects with paranoid schizophrenia, but not in those with the diagnosis of residual schizophrenia, which is consistent with the volumetric results described by Cascella et al. (61).

One possibility which should be explored in future studies is that the insula and claustrum may act in concert, or may both be components of a larger network associated with salience processing or “reality testing.” This network would likely include other subcortical nuclei such as the amygdala, which also has widespread connectivity with the medial prefrontal areas and anterior cingulate cortex, and which (along with voxels in the insula-claustrum region) exhibits increased activity associated with referential ideation in people experiencing delusions (65). Strong reciprocal connectivity between the anterior claustrum and the amygdala (as well as the insula) has been shown in rats, and seizures induced by kindling in the anterior claustrum resemble seizures generated in the amygdala (66). Damage affecting the claustrum, insula, or amygdala, alone, or more likely, in concert, could lead to or potentiate delusional ideas by disruption of functional connectivity in such a network.

## Claustrum Lesions

An obvious approach to examining a putative link between claustrum function and the presence of delusional thinking is assessment of the relationship between the occurrence of delusions and damage to the claustrum in said patients (see Table 2). However, spontaneous lesions restricted to the claustrum are exceedingly rare. Developmentally, agenesis of claustrum is associated with other severe brain malformations, with poor prognoses (67, 68). Isolated bilateral claustrum lesions in adults are similarly rare, and we found only two in the literature, one viral (58) and one non-viral (59) transitory encephalopathy. In both cases, patients presented with psychotic symptoms.

**Table 2:** **Case reports and individual findings from congenital, spontaneous, and iatrogenic claustrum lesions**.

Reference	Claustrum Lesions	Other affected brain areas	Diagnosis	Claustrum lesion mechanism	Psychiatric and non-psychiatric symptoms	Evolution of psychiatric symptoms	Notes
Dodgson (67)	Absence of dorsal claustrum	Bilateral insular microgyria, abnormal frontal and temporal sulci adjacent to the insula	Mental retardation	Brain malformations	Mental retardation	Not commented upon	
Ishii et al. (58)	Bilateral claustrum	Nil	Viral (mumps) encephalitis	Edema Inflammatory?	Confusion, visual and auditory hallucinations, epilepsia	Reversible	
McKay and Cipolotti (69)	Right claustrum	Right insula, adjacent white matter, less severe changes in left insular cortex	Herpes simplex encephalitis	Edema Inflammatory? Immune reaction?	Cotard delusion status epilepticus	Reversible	
Sperner et al. (59)	Bilateral claustrum	bilateral External capsuale	Transitory non-viral encephalitis	Edema Inflammatory?	Psychotic symptoms	Reversible	
Shoji et al. (70)	Bilateral claustrum	Both hippocampi, both amygdalae	Non-herpetic acute limbic encephalitis	Edema Inflammatory? Immune reaction? CSF positive for anti-GluRϵ2 IgG and IgM antibodies	Delirious state, restlessness, palpitation, seizures	Reversible	Patients with non-herpetic acute limbic encephalitis (NHALE) often manifest behavioral disorders, incoherent speech, delusions and hallucinations. This it to put the presence of auto antibody against glutamate receptor in NHALE could lead to a malfunctioning glutamate systems and then the disruption of dopaminergic pathways, as suggested in the glutamate model of delusions
Ishida et al. (71)	Bilateral claustrum	Right hippocampus	Non-herpetic acute limbic encephalitis	Edema Inflammatory? Immune reaction? CSF positive for auto antibody against glutamate receptor	Headache, convulsion, consciousness disturbance, ataxia, cold-like symptoms. disturbance of short-term memory and a change of character	Reversible unless memory disturbances	
Matsuzono et al. (72)	Bilateral claustrum	Medial of frontal lobe, periventricular region	Non-herpetic acute limbic encephalitis	Edema Inflammatory? Immune reaction? CSF positive for auto antibody against glutamate receptor	Delusional ideas and hallucinations, but not seizures (personal communication to CP) Parkinsonism, myoclonus	Reversible	
Chakraborty et al. (73)	Left claustrum	Multiple cortical (insular, medial and lateral frontal cortex), and periventricular (caudate head) discrete ring enhancing lesions and associated surrounding edema	Multiple parenchymal neurocysticercosis	Oedéma Inflammatory?	Delusion of jealousy left-sided hemiplegia	Reversible	
McMurtray et al. (74)	Left claustrum	Left basal ganglia with adjacent edema likely affecting the corona radiate and possibly extending to the optic radiations	Hemorrhagic stroke	Necrosis?	Neurological impairment, visual and auditory hallucinations, and delusions of rotting/decaying of the right (paralyzed) side of his body similar to a Cotard delusion	Reversible with antipsychotic medications	
		Small periventricular hyperintensities		Perinecrotic edema			
Turkalj et al. (75)	Left claustrum	A 10 cm tubular area of posttraumatic encephalomalacia of the left hemisphere (left orbitofrontal region, insula, putamen, deep white matter and parietal lobe with consecutively slightly enlarged left lateral ventricle)	Stabbing injury from a billiard stick	Necrosis? Post traumatic gliosis Edema?	Delusions with paranoid and religious content accompanied by visual hallucinations, anosognosia, bradypsychia, anhedonia, depressed mood disinhibited behavior, and progressive social withdrawal, left eye mydriasis	Reversible	
Sener (76, 77)	“Bright claustrum sign” (T2 claustrum hyper intensity)	–	Wilson’s disease (WD)	Oedéma Inflammatory?	Neurological symptoms, no remarks about psychiatric symptoms	–	Delusional disorders and schizophrenia-like psychosis have also been associated with WD (78–81)

In other clinical cases presented in the literature (see Table 2), the claustrum is far from being the only affected cerebral structure. For instance, in cases of non-herpetic acute limbic encephalitis (NHALE) with bilateral claustrum lesions, patients experienced psychotic symptoms (70, 72), the claustrum damage occurred alongside lesions of other limbic structures. This precludes inference of a causal role for the claustrum in the observed symptoms, including delusions, where present, but offers guidance regarding which structures may present useful experimental targets for further exploration.

Somewhat surprisingly, Duffau et al. (82) reported that in a relatively large cohort of patients (*N* = 42), unilateral removal of the claustrum (and typically the overlying insula) did not generally produce sensory, motor, or cognitive deficits, and the majority of patients returned to near normal function (although criteria for normal function were not detailed). This suggests that there is some compensatory capacity between the claustrums of different hemispheres, though the extent of compensatory function remains uncertain, and will require more specific experiments. In subsequent sections, we will consider the available research data surrounding the functional organization of the claustrum, and its potential roles in normal and pathological brain function.

## Hypotheses Regarding Putative Mechanisms for Claustrum (DYS)Function in Delusions

Over the last 30 years, explanations for psychiatric pathologies in general and the genesis of delusions in particular, have migrated from philosophical, to biological, and subsequently to neurocomputational models. Although there are strongly suggestive radiological or anatomical data that implicate claustrum dysfunction in delusions (61, 62, 64), a compelling theoretical model for a biochemical or physiological mechanism of claustrum involvement in delusional pathologies has yet to be elucidated. Several candidate neurochemical pathways are outlined below, as well as possible neurocomputational mechanisms.

## Dopamine and Glutamate Model

Current pharmacological and neurochemical models of delusions include the dopaminergic (DA) and glutamate pathways. The DA model explains why drugs currently used in treatment for patients with delusions, independent of causal pathology, are mainly dopamine antagonists which act *inter alia* at the level of D2 receptors on the mesolimbic DA pathway. The glutamate model suggests that malfunctioning glutamate systems lead to disruption of DA pathways, acting as an upstream agent in the pathophysiology of delusions (83, 84).

Although not well studied, there are some apparent links between the claustrum and the pharmacological model of delusions (e.g., the glutamate model and mesolimbic DA pathway; Box 2). As alluded to previously, some patients suffering from NHALE present with bilateral claustrum lesions and such psychotic symptoms as hallucinations and delusions (70, 72). Furthermore, NMDA receptors are moderately expressed in the claustrum of New World monkeys (85) and strongly expressed in rat and human claustrum (86).

Box 2 **Dopamine and glutamate models of delusions.**

At a psychopharmacological/neurotransmitter level, there are two main theories thought to account for delusions: the dopaminergic and glutamate models. The DA theory explains why drugs currently used in treatment for patients with delusions, independent of the causal pathology, are mainly DA antagonists which act *inter alia* at the level of D2 receptors on the mesolimbic DA pathway (MLDA). The glutamatergic model suggests that malfunctioning glutamate systems impacts the dopamine system and thus plays a critical, but indirect, role in the pathophysiology of delusions.

Glutamatergic modulation of the dopamine pathway is thought to be mediated via pyramidal cells in the prefrontal cortex (PFC) and ventral hippocampus, projecting locally on parvalbumin inhibitory interneurons (PV-IN) via glutamate NMDA postsynaptic receptors. The PV-IN, in turn, inhibits pyramidal cells via GABA-A receptors (83, 87). Insufficient inhibition of PFC pyramidal cells has been suggested as a pathway for excessive release of glutamate in the ventral tegmental area (VTA), leading to excess dopamine release in nucleus accumbens (88). Mesolimbic dopamine hyperactivity is implicated in delusions (89–91), as evidenced by the observation that phencyclidine and ketamine, both of which are known to cause delusions (92), affect glutamatergic neurotransmission in PV-IN of the PFC (93, 94). The glutamate model is further supported by the nearly uniform co-occurrence of delusions with NHALE (95), an encephalitis characterized by the presence of anti-glutamate (NMDA) receptor antibodies in the blood and/or the cerebrospinal fluid, and in which delusions are present in 90% of diagnosed patients.

Direct DA modulation of claustrum cells is possible but does not appear to be a dominant influence based on the neurochemical architecture. Receptor autoradiography has shown DA receptors (D2-type) are present at light-moderate levels (~3% of putamen strength) in the claustrum in postmortem human brains (96), and moderate-heavy expression of D2 mRNA has been reported in the rhesus monkey claustrum (97). Other DA receptor subtypes have not been reported at significant levels in the primate claustrum. However, the claustrum has anatomical connections with most of the components of the mesolimbic DA system and could modulate this pathway via excitation or indirect inhibition of mesolimbic pathway components at multiple levels. For example, the claustrum receives projections from the VTA (33) and projects to the nucleus accumbens (33, 66, 98).

## Kappa Opioid Receptor Model

A better candidate for DA modulation of the claustrum may be found in the kappa opioid receptor (KOR) system. Kappa opioid receptors are densely expressed in the claustrum of rodents (99), Old World (100, 101) and New World (102) monkeys, and humans (103, 104), and it has been suggested that the hallucinogenic, delusionogenic, and psychotomimetic effects of the kappa opioid agonist salvinorin-A may be due to over-activation of the claustrum kappa receptor population (105). Much work remains to be done with respect to the psychopharmacology of the claustrum, and it should be noted that there is some evidence that salvinorin-A effects could be partially mediated by non-opioid specific activation of, e.g., the cerebellum (106).

Exploration and elucidation of claustrum effects on various transmitter systems and consequent influence on psychotic symptoms will require increased awareness of the claustrum on the part of researchers and clinicians, and design of studies to specifically monitor the claustrum in psychiatric conditions. Enhancement of this awareness is a key objective of this review.

## Sensory Gating and Attention Model

Sensory gating is the ability to filter out irrelevant information from environmental stimuli, and thus reduce the load of redundant or unnecessary information in the higher cortical areas. Sensory gating is generally studied using paired pulse inhibition, which compares the P50 components of the event-related potentials from two successive auditory stimuli (clicks) separated by 500 ms. Under normal conditions, P50 amplitude is reduced in response to the second stimulus, and this is attributed to adaptation by sensory gating. In a combined EEG and fMRI study, Bak et al. (107) suggested that the claustrum, together with the hippocampus, mediates the inhibitory processes of P50 suppression. In neurologically healthy human subjects, P50 amplitude decreased by 80–90% following the second pulse, whereas amplitude is reduced by only 10–20% in subjects with schizophrenia (108, 109). Reduced sensory gating is also seen in other psychotic processes (108), and is implicated in the formation of psychotic symptoms via changes in latent inhibition. Altered latent inhibition, in turn, is a predictor of medication efficacy for positive symptoms of psychosis (8, 110, 111).

A similar paired-pulse inhibition (PPI) assay is widely used as an animal model for psychotic symptoms [reviewed in Forrest et al. (8) and Ratajczak et al. (112)]. Bortolato et al. (113) and Ruderman et al. (7) have worked on rat and mouse models, respectively, of psychosis induced by subcutaneous injection of selective KOR agonists, which disrupt acoustic PPI. In those experiments, PPI suppression was reversed by pretreatment with KOR antagonists, and also by the atypical (antiserotonergic and antidopaminergic) antipsychotics clozapine and AC90179 (a highly selective 5-HT2A receptor inverse agonist). Reversal was not observed with pretreatment using the antidopaminergic haloperidol. We are unaware of any studies that have specifically examined PPI-like effects in claustrum responses using animal models, although the time scale of claustrum responses to auditory stimuli in non-human primates overlaps the PPI interval (114), so such an experiment may be feasible in the future.

Sensory gating is also a key component of early attentional modulation and stimulus salience. Remedios et al. (114, 115) have suggested that the claustrum could be a detector of novel or salient sensory events, and this and other observations have led Goll et al. (27) to propose that one fundamental role of the claustrum may be modulation of selective attention. The interested reader is referred to their comprehensive review and analysis of the attention hypothesis for more information. This is an area ripe for experimental investigation, and future genetic knockout or transfection models will likely help to clarify the role of the claustrum in psychosis and sensory gating.

## Claustrum as a Component of Behavioral and Psychological-Level Models of Delusions

Two main characterizations of delusions as a psychological/behavioral phenomenon have been proposed in recent years: (1) delusions as an error of predictive modeling or validity testing of external stimuli, and (2) delusions as abnormalities of stimulus salience. The widespread connectivity and polymodal nature of the claustrum is consistent with a role in either of these models, as described below.

Llinas and Roy (116) have modeled the mammalian brain as a Bayesian prediction engine. In this construct, behavior during normal function includes generation of predictive models of motor outcomes. Llinas and Roy suggested that these predictive models act to compare incoming sensory signals against internal, learned representations of the external world. This system allows feedback analysis of the effects of motor actions (the outward manifestations of behavior), as well as predictions regarding possible outcomes of motor action. Learned representations form the prior knowledge required for a Bayesian system, and as new observations occur, previous judgments are reviewed, giving an increasingly low weight to the prior beliefs. This has the effect of minimizing the prediction error.

Under the model of Llinas and Roy (116), sensory-motor representations are encoded in the oscillatory dynamics of the thalamocortical circuit, and individual variations in thought and behavior could exist as stochastic variances in oscillatory behavior across thalamocortical networks. In several key ways, the claustrum offers a more plausible locus for this activity. Claustrum-cortex connectivity offers a relatively uniform “path length” between diverse areas of the cerebral cortex (21), and the sheet like and elongated geometry of this structure may facilitate communication between, e.g., prefrontal areas and parietal and occipital regions of cortex. Moreover, the thalamocortical oscillatory model necessitates a functional connection between modality specific areas, e.g., primary visual cortex (V1) and lateral geniculate nucleus (LGN), with polymodal information mediated through the “non-specific” nuclei of the thalamus (117, 118). In contrast, Pearson et al. (32) have demonstrated that regions of cortex that are interconnected, even if they do not share the same sensory modality or internal vs. external representation, exhibit overlapping patterns of structural connectivity in the primate claustrum. This suggests that rapid, polymodal, internal vs. external computations could be efficiently mediated by the claustrum. Corlett et al. (119–121) have suggested that delusions arise from responses of brain circuits to prediction errors. Thus, in delusional patients, bottom-up signals (new information brought by external sensory experiences) are aberrant, indicating to the patient that prior beliefs are false and should be adjusted to explain the world. In this case, the patient does not take past experiences appropriately into account. Finally, whereas lesion studies are problematic at the behavioral/psychological level for the reasons outlined in previous sections, it is interesting to note that lesions of the thalamus are not generally associated with psychotic symptoms, in contrast to the lesions of the claustrum–pulvinar–insula region described above.

Gandola et al. (12) performed a lesion-mapping analysis of cases of somatoparaphrenia (delusional belief that a paralyzed limb or side of the body belongs to another individual) and identified a region of maximum overlap across patients that involved both a portion of the lateral thalamus, as well as the claustrum–pulvinar–insula region. Yet again, these authors did not specify damage to the claustrum, although it is implicit in the published figures, and implies damage to the adjacent white matter tracts as well. Thus, it is impossible to state conclusively whether damage to either thalamocortical or claustrum-cortical circuits in isolation could result in delusions. We predict that this ambiguity will be resolved as the anatomy and centrality of connections of the claustrum become more widely known to investigators and clinicians.

The second major psychological/behavioral model of delusional thinking is the suggestion by Coltheart (122) and Coltheart et al. (123), among others, that delusions result from an imbalance between the production of false beliefs (possibly resulting from a disturbance of stimulus salience by increased mesolimbic dopamine activity) and the inability to assess the validity or reality of these beliefs. They predict that the main cortical loci of this reality testing resides in the right PFC [reviewed in Coltheart et al. (123)]. Recent connectivity studies in non-human primates have identified a dense, overlapping pattern of connections between several areas of the dorsolateral PFC and the rostral and mid-ventral levels of the claustrum (23, 124). This circuit provides a plausible anatomical substrate for the reality testing model, and will, for the first time, provide a means of targeting specific regions of the claustrum for experimental manipulations and high resolution imaging. This, in turn, will facilitate experimental examination of hypotheses related to a role for the claustrum in the schema described above.

## Conclusion

Here, we have summarized the suggestive, but not conclusive, body of evidence that implicates the claustrum in the underlying pathology of delusional thinking. We consider this report a successful foray if clinicians, in particular those who concentrate on patient populations where delusions are common, incorporate the claustrum and the possibility of claustrum damage into their understanding of the disease processes. Several hypothetical functions of the claustrum have been proffered, any of which could affect delusional pathologies. It is of course possible that these suggestions could be rendered moot should ongoing research identify an unrelated function of the claustrum in normal and/or pathological behavior. However, the consistency of delusions as an accompanying feature of such a wide range of syndromes in which structural changes in the claustrum have been reported suggests strongly to us that this is a necessary and potentially quite fruitful line of inquiry for future studies. Parallel advances in understanding the basic science of the claustrum, including its neurochemistry, cytoarchitecture, and functional connectivity, will likely yield potential therapeutic targets, including both surgical and pharmacologic approaches for ameliorating disease associated with alterations of claustrum function. We suggest here that delusions may represent an outward manifestation of one such disease process, secondary to a structural or functional disturbance of information processing of the claustrum.

## Conflict of Interest Statement

The authors declare that the research was conducted in the absence of any commercial or financial relationships that could be construed as a potential conflict of interest.
